# How to do an electrophysiological study of tremor

**DOI:** 10.1016/j.cnp.2019.06.002

**Published:** 2019-06-28

**Authors:** Felipe Vial, Panagiotis Kassavetis, Shabbir Merchant, Dietrich Haubenberger, Mark Hallett

**Affiliations:** a Human Motor Control Section, National Institute of Neurological Disorders and Stroke, National Institutes of Health, Bethesda, MD, USA; b Facultad de Medicina Clínica Alemana Universidad del Desarrollo, Santiago, Chile; c Department of Neurology, College of Medicine, Medical University of South Carolina, Charleston, SC, USA

**Keywords:** Tremor, Electrophysiology, EMG, Accelerometry

## Abstract

The electrophysiological characterization of hand tremors is a useful method to complement the history and physical exam of tremor patients. Our article describes the methodology (recording, processing and interpretation) used in a diagnostic/phenotypic hand tremor study conducted in our lab at the Human Motor Control Section of the National Institute of Neurological Disorders and Stroke (NINDS), at the National Institutes of Health. The necessary equipment includes two one-axis accelerometers and four-channel electromyography (EMG). The hand tremor is recorded at rest, posture with and without weight loading, and during movement (kinetic). The recorded signals are analyzed in the time and frequency domains. The characterization of the dominant frequencies in the accelerometers and their relationship with the EMG frequencies are essential for the differential diagnosis of different tremor syndromes. We describe the electrophysiological characteristics of several tremor syndromes such as enhanced physiological tremor, essential tremor, Parkinson tremor, pharmacological-induced tremor, orthostatic tremor, and functional (psychogenic) tremor. Simplified guidance for adoption of tremor studies as a clinical tool in a movement disorders subspecialty clinic is provided.

## Highlights

• An electrophysiological study of tremor can be helpful for the diagnosis.
• A study of hand tremor can be done with surface EMG and an accelerometer.
• Analysis in the frequency domain allows separating the different tremor components.
• Coherence analysis shows whether there are one or more oscillators.

## 1 Introduction

The importance of tremor phenomenology is emphasized in the consensus criteria on tremor classification by the International Parkinson and Movement Disorder Society published in 2018 (Bhatia et al., 2018). Specifically, tremor phenomenology corresponds to the first axis in this classification, which includes clinical characteristics, historical features, associated signs, imaging and electrophysiology. This combined information from the first axis may be suggestive of one or more etiologies, which correspond to axis two in this tremor classification. In addition to reflecting the importance of appropriate characterization of tremors based on phenomenology, the consensus statement also refers to the limitations of this approach which is based solely on clinical assessment. The limitations of clinical assessment are clear from high rates of misdiagnosis (upwards of 37%) of essential tremor syndrome, which is the most common (Jain et al., 2006).

Tremor analysis provides objective, reproducible, and diagnostic information about tremors. The diagnosis of certain tremor syndromes such as orthostatic tremors can only be made using objective physiology (McManis and Sharbrough, 1993). Additionally, the electrophysiological characterization of tremor is useful to complement the history and physical exam in tremor patients. It can provide not only information about tremor frequency, but also inform the clinician about the presence of mechanical, mechanical-reflex, or central components of the tremor. In addition, the electrophysiological characterization of the tremor makes it possible for the clinician to differentiate between tremors produced by one or multiple oscillators. Finally, it can be very useful to uncover a functional tremor syndrome and serve as an objective diagnostic test which can have prognostic implications (Schwingenschuh and Deuschl, 2016).

Our review describes the methodology (recording, processing and interpretation) used in a standard diagnostic/phenotyping tremor study conducted at the Human Motor Control Section of the National Institutes of Neurological Disorders and Stroke (NINDS) at the National Institutes of Health. We focus on the study of hand tremor, as it represents the body part most commonly affected by tremors. However, the same methodology with minor adjustments can be used for studying tremor in any other body part. This review focuses on tremor measurements that, from a practical point of view, may be most useful in a clinical setting.

## 2 Methods

To characterize tremor, we record the movement itself and the activity of the muscles that may be producing the movement.

The trajectory of a movement of any object in space has 6 degrees of freedom; 3 for translations in a 3-dimensional space, and 3 for rotations. Therefore, in order to capture all the movement parameters observed in a tremor, a triaxial accelerometer plus a triaxial gyroscope are needed (Elble and McNames, 2016). Historically, accelerometers have been used more frequently than gyroscopes for tremor analysis and over the last 50 years, extensive experience has been accumulated on their use (Elble and McNames, 2016).

The methods we describe includes the use of a 1 axis accelerometer, which is more practical as it only requires low-cost equipment that is often available in the clinical setting. Otherwise, a 16-channel amplifier would be necessary: 3 channels for 3-axial accelerometry, 3 channels for 3-axial gyroscope, and two electromyography (EMG) channels for each limb. However, use of the one-axis accelerometer is a compromise as this method cannot capture all the parameters of the movement, is susceptible to gravitational artifacts, and relies on the correct placement of the accelerometer in such a way that the axis measured is aligned with the main axis of the motion.

Surface EMG is used to measure the muscular activity which is the second parameter. We always place surface EMG electrodes in pairs, covering both the agonist and antagonist muscles that are participating in the movement. For example, for the study of hand tremor with a predominant flexion-extension movement at the wrist, we place the electrodes on the wrist flexors and extensors muscles on the forearm. The ground electrode is usually placed on a bone prominence (e.g., olecranon).

### 2.1 Recording hand tremor

The patient is seated in a comfortable chair with both forearms pronated and resting on the armrests leaving the hands free in the air to measure the hand tremor. This position is crucial in order to isolate as much as possible the hand tremor from any other body movement that could potentially contaminate the signal of the accelerometers. The area of the skin where the electrodes are placed is thoroughly prepared in order to reduce possible artifact and reduce impedance (goal <10 KΩ) (Hermens et al., 2000). It is important to place the surface EMG electrodes with the arm in the position of the recording since the relative position of the skin and underlying muscles change with forearm rotation. The two electrodes are placed 2–4 cm apart in the longitudinal axis of the muscle. The accelerometers are secured with a band on the dorsum of the hand with the recording axis aligned to the dominant tremor axis, which is usually in the vertical plane.

In regard to the amplifier set up, the sampling rate should be at least 4 times the highest frequency of interest. Considering that the frequency content of the EMG signal can be as high as 250–300 Hz, a sampling frequency of at least 1000 Hz should be used (Nilsson et al., 1993). For the accelerometers, a 2 Hz highpass and a 30 Hz lowpass filter is used, and for the EMG a 10–20 Hz highpass and a 250 Hz lowpass filter is used. These filters can be set for online processing during acquisition or can be wider and applied offline.

The tremor is recorded during rest, posture, and kinetic action. Usually, a recording time of 30–60 s provides sufficient data to characterize the tremor (Elble and McNames, 2016). Longer recordings may induce fatigue in the patient particularly during posture or action, which is a concern.

During the recording of the rest tremor, the forearm and hand must be fully relaxed while the forearms are supported on the armrest and the hands are suspended freely in the air, without touching the chair. During the posture recording, the patient is instructed to extend the wrist against gravity while keeping the pronated forearm resting on the armrest, with the hand extended beyond the edge of the armrest. To separate peripheral from central tremor components, the postural tremor is recorded with and without weight loading (in our lab we use 1, 1.5 and 2 lb). Throughout the recording with weight loading, the weights are attached to the dorsum of the hand. For the action tremor recording, the patient is instructed to slowly and continuously flex and extend the hand at the wrist. It is important to emphasize to the patient to perform this movement slowly (<2 Hz), so the low frequency registered from this voluntary movement can be recognized and separated from the tremor frequency.

### 2.2 Processing the data

The acquired data will be a time series showing change of voltage over time in the case of the EMG or change of acceleration over time in the case of the accelerometer. When the data are in a format of change over time, this is the “time domain”, and although most of the tremor analysis is done on the basis of the “frequency domain” (which will be explained below), it is important to review the data in this format to make sure that it is clean from artifacts, to observe agonist-antagonist muscle interaction, burst duration, and the presence of abrupt changes that may affect further analysis in the frequency domain.

The tremor by definition is periodic, therefore it has a particular frequency, which is defined as the number of oscillations per unit of time. The frequency can be manually extracted from the time domain signal by counting the number of cycles per one second, but this is not a very precise method and can be very challenging in a signal like EMG which is composed of many frequencies. Thus, a better approach to study the frequencies is to transform the signal from the time domain to the frequency domain, usually done with a Fourier transformation (Hallett, 1998).

**Fig. 1:**
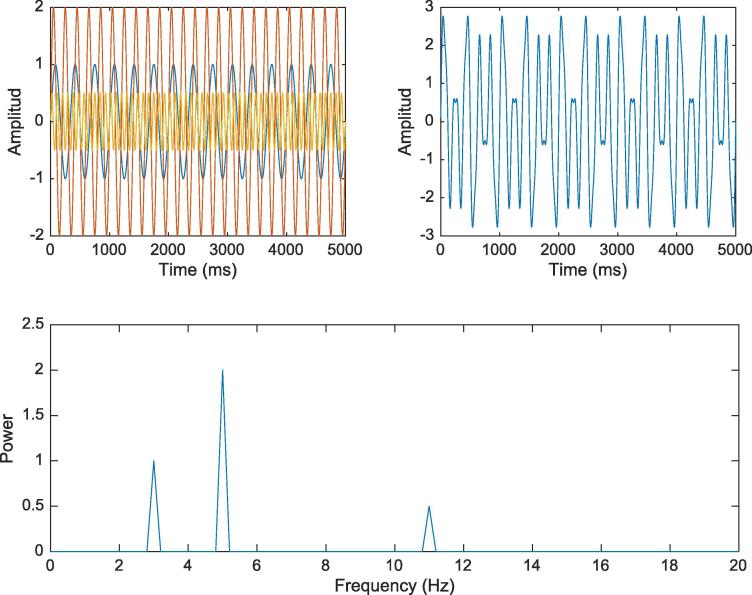
Moving from the time domain to the frequency domain. The first plot shows 3 different sine waves (3, 5, 11 Hz) with different amplitudes overlapped over time. The second plot shows the result of the sum of the 3 sine waves. The plot on the bottom is the frequency representation of the second plot after a Fourier transformation.

A Fourier transformation analysis consists of a series of convolutions between the studied signal and sine waves of different frequencies. The result between the convolution of the signal and a sine wave of a specific frequency is a dot product, whose magnitude provides an idea of how “important” that frequency is within the signal. The magnitude is expressed as power which mathematically is the amplitude squared. The results of each convolution are plotted in a frequency by power plot which provides information about the relative power of the different frequencies within the recorded signal (Cohen, 2014) (Fig. 1).

The number of sine waves used for the Fourier transformation is equal to the number of sampling points of the studied signal, and the frequencies of the sine waves range from 0 to half of the sampling rate (i.e., the Nyquist number). Therefore, the maximum number of frequencies that can be studied depends on the sampling rate, and the frequency resolution depends on the number of points in the segment.

The Fourier transformation analysis assumes that the signal is stable over time. If there are important changes of the tremor’s characteristics over time, it is recommended to segment the data into small pieces within which the tremor is stable. Then run the Fourier transformation on each segment separately. Two important things must be considered with this approach; first, the frequency resolution will now be determined by the length of the new segments; and, second, a taper (e.g., a Hanning window) must be applied on each segment before the Fourier analysis in order to avoid edge artifacts (artifact that arises from the beginning and end of the signal when it is transformed into the frequency domain). The results of the Fourier transformation analysis of all the segments can be averaged and expressed as a power spectral plot, which shows the change in the power of each frequency over time.

Before the data’s conversion to the frequency domain, the EMG data must be rectified (transform each point to its absolute value) and smoothed in order to emphasize the frequency of the EMG bursts as opposed to the EMG activity within a burst and to increase the signal to noise ratio (Milner-Brown and Stein, 1975).

Another type of analysis that may be done is to look for coherence (or magnitude of the squared coherence) between two channels. Coherence analysis expresses the similarity in frequency of two signals. It is equivalent to a Pearson correlation in the frequency domain, and it is obtained by dividing the cross spectrum of both signals by the autospectrum of each one of them (Halliday et al., 1995). The values obtained for each frequency run from 0 to 1, where 0 is no coherence and 1 is perfect coherence.

## 3 Interpretation of the data

### 3.1 Background

In order to read the data of a tremor study, it is important to understand the concept of natural frequency, which is that any object will oscillate at a given frequency when receiving energy. The object’s frequency of oscillation will depend on its physical properties according to the following formula:

$$w=\surd \left(K/l\right)$$

The natural frequency (w) depends on the square root of the object’s stiffness (K) divided by the object’s inertia (I), which is driven by its mass (Hallett, 1998).

Each part of the body, because of its physical properties, will unavoidably oscillate at its natural frequency when not restrained. This oscillation is the mechanical component of tremor and is recognized by the change in frequency during weight loading (note that weight loading affects the inertia which is a factor in the above formula) (Deuschl et al., 2001, Hallett, 1998). The energy for this tremor component comes from irregularities in the firing rate of motor units and the force produced by the cardiac systole, the ballistic cardiac impulse (Elble and Randall, 1978, Marsden et al., 1969). Under certain circumstances in which the gain of the monosynaptic stretch-reflex is increased (e.g., fatigue, stress, intake of adrenergic medication), the muscle stretch produced by the mechanical component may trigger a reflex response that can exacerbate the mechanical component which is then called the mechanical-reflex component (Hallett, 1998, Elble, 1996).

A tremor may also originate from one or more structures in the central nervous system experiencing an aberrant oscillatory activity that is transmitted along the motor system (Hallett, 1998). In this situation, the tremor is considered to be of central origin, and its frequency does not change when weight is added. The presence of one or more oscillators participating in the generation of tremor is recognized by comparing the frequencies of oscillations in different limbs with coherence analysis. If all the limbs are oscillating at the exact same frequency, this is evidence for a single oscillator. However, if the frequencies are different across limbs, one may assume that there are several independent oscillators. As will be further discuss, the identification of one vs multiple oscillators causing the tremor can be very important for the diagnosis.

### 3.2 Analysis

Once the data is transformed to the frequency domain, further analyses may be conducted. The idea is to look for the different components that may be participating in the generation of the tremor, namely, mechanical component, mechanical-reflex component, and central component. Additionally, coherence analysis may be run between channels to compare the components in the frequency domain.

The first step is to describe the main peak (or peaks) in the accelerometry spectrum (i.e., frequency domain) which represents the frequency of the tremor. If the same frequency peak can be found in the EMG frequency spectrum of a specific muscle, then there is EMG correlation of the tremor, and this means that this muscle is participating in the generation of the tremor. If there is no peak in the EMG at the same frequency as in the accelerometer, the tremor is likely not driven by this muscle’s activity. This is indicative that the tremor is purely mechanical (or caused by the activity of a muscle that was not recorded). In some cases, it may be difficult to see if the accelerometry and the EMG peaks have the same frequency; this can be solved by running coherence analysis between the two channels and looking for a common peak.

**Fig. 2:**
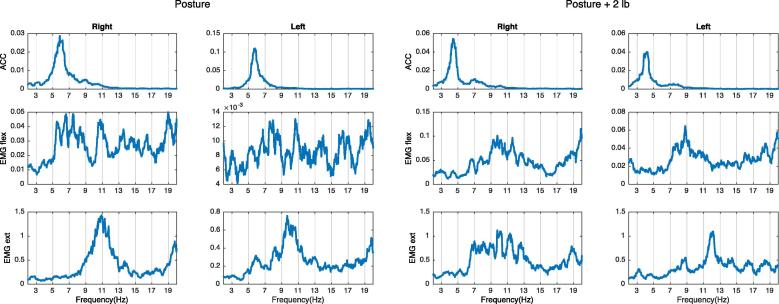
Example of mechanical tremor component. There is a peak on the right-side accelerometer at around 6 Hz that decreases in frequency after weight loading, and it does not have a clear EMG correlation. This is compatible with a mechanical tremor component.

Next is to determine if there is any change on the frequency peaks after weight loading. If the peak on the accelerometer decreases in frequency more than 1 Hz after adding weights (Elble, 2003), this peak is caused by a mechanical oscillation as it follows the rules of the natural frequency (Fig. 2).

**Fig. 3:**
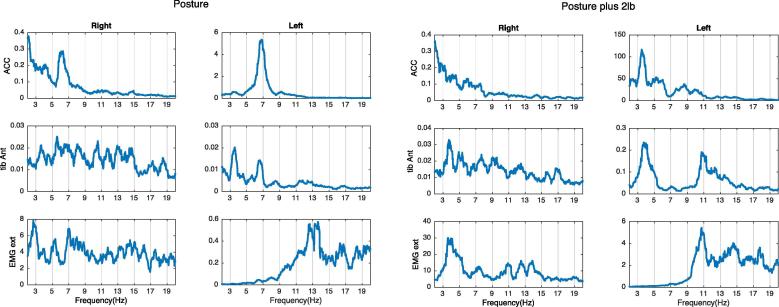
Example of mechanical reflex tremor component. There is a peak on both the accelerometer and the EMG at around 7 Hz on the left hand. After adding weight, peaks on the ACC and the EMG both shift to the left. This pattern is compatible with a mechanical-reflex component.

If a peak in the EMG spectrum, with a corresponding peak at the same frequency in the accelerometer spectrum, decreases in frequency after weight loading, that means that the limb is oscillating at its natural frequency and the oscillation is increased by a short loop spinal reflex, and the two oscillations are mutually entrained (see below). This frequency peak is then considered to represent a mechanical-reflex component of tremor (see example in Fig. 3).

**Fig. 4:**
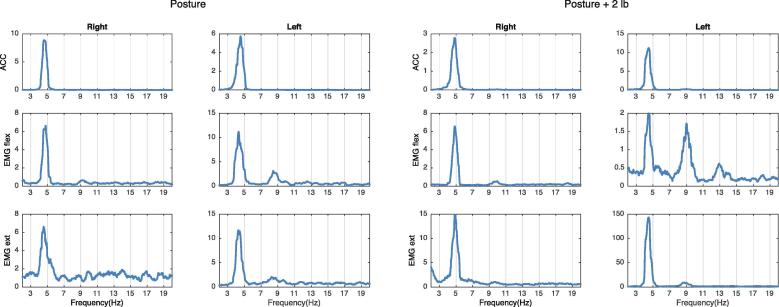
Example of central tremor component. There is a 5 Hz peak on the right ACC and an EMG that does not change frequency with weight loading. This is typical for tremors driven by a central oscillator.

If there is a peak in the EMG spectrum at a similar frequency as in the accelerometer spectrum, and none of the peaks change when weight is added, the limb is not oscillating at its natural frequency and therefore a central tremor oscillator is presumed (Fig. 4).

**Fig. 5:**
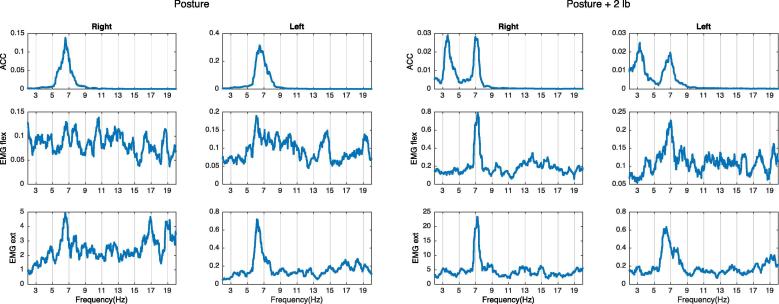
Example of mechanical and central component. There is a bilateral 6–7 Hz peak on both the ACC and EMG. After weight loading, the peak splits into a central and a peripheral component.

**Fig. 6:**
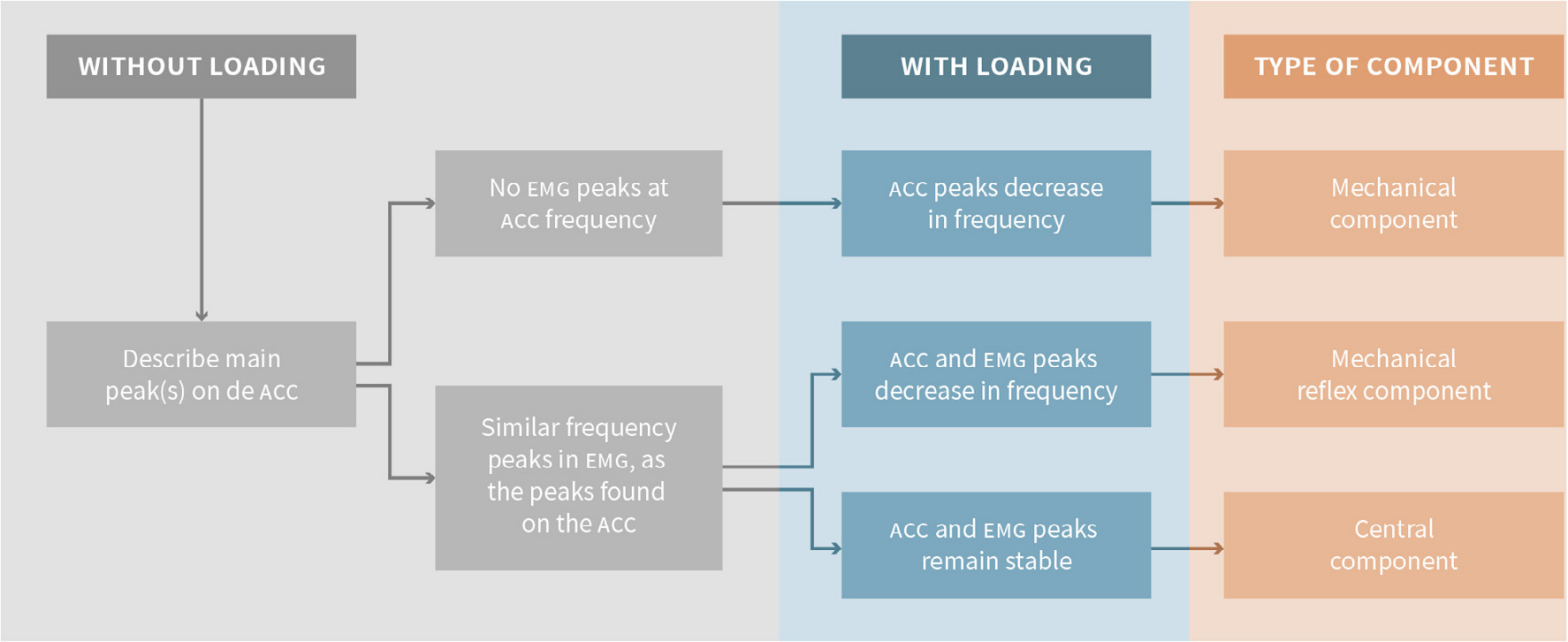
Flow chart to analyze a tremor study. Summary of the steps to analyze a tremor study in the frequency domain.

It is possible to have in the same limb more than one component in a tremor. When two components are very close together in the frequency domain, they may fuse into one peak because of resonance (e.g., when there is a mechanical and a central component resonating). In this situation, it is possible to separate the components by adding weights (Fig. 5). Fig. 6 includes a summary of the abovementioned possibilities.

Additionally, coherence analysis may be run between left and right EMG channels to define the number of oscillators participating in the tremor. As will be further discussed, this is relevant for the diagnosis of functional (psychogenic) tremor and orthostatic tremor.

### 3.3 Clinical interpretation

After the analyses, the results must be interpreted as part of a clinical syndrome. In the following section we describe the possible findings in the most common tremor syndromes.

#### 3.3.1 Physiological tremor

In physiological tremor, there is usually only a mechanical component with no EMG correlation. In the hand, the frequency of the mechanical component typically ranges between 6 and 12 Hz. Raethjen and colleagues also report in a study on 117 healthy voluntaires a central component participating in the tremor in the 8–12 Hz band (Raethjen et al., 2000). The mechanical component usually has a higher amplitude than the central component in the accelerometer (Elble, 2003).

#### 3.3.2 Enhanced physiological tremor

The frequency of enhanced physiological tremor ranges from 4 to 12 Hz and up to 3 components may be seen: a mechanical component, a mechanical-reflex component, and an 8–12 Hz central component (Deuschl et al., 2001, Haubenberger and Hallett, 2018).

#### 3.3.3 Essential tremor

In essential tremor it is common to find a 4–12 Hz bilateral central component plus a mechanical component, and in many cases the components have a similar frequency and will merge (Haubenberger and Hallett, 2018). With weight, the mechanical component can be separated.

The frequency of essential tremor is higher in young people as there is an inverse correlation of essential tremor frequency and age (Calzetti et al., 1987). When tremor frequencies are found in the 8–12 Hz range the differential diagnosis between essential tremor and physiological tremor can be difficult.

#### 3.3.4 Pharmacologically induced tremor

Many drugs of different classes may cause or enhance tremor. In particular, adrenergic drugs are known to increase the mechanical-reflex component by increasing the gain of the γ-loop (Morgan and Sethi, 2005). Another study reported that amitriptyline induced tremor by enhancing the central component (Raethjen et al., 2001). In addition, drugs that block dopamine receptors may cause a parkinsonian tremor (Caligiore et al., 2016). The list of drugs that can induce tremor is extensive. However, a detailed electrophysiological characterization of tremors that may be caused by drugs is not available at this time.

#### 3.3.5 Parkinson tremor

In Parkinson disease, most commonly there is the classical 4–7 Hz “rest” tremor that may also “re-emerge” in posture (Bhatia et al., 2018), but is also possible to find a separable postural tremor. In both cases, the main component is central.

In regard postural tremor, Dirkx et al. found that it was present in 82% in a group of 77 patients who also had rest tremor. Furthermore, with cluster analysis they were able to sub-divide the postural tremor into re-emergent tremor and pure postural tremor. Re-emergent tremor was 81% of the postural tremors. The tremor paused for several seconds after the assumption of posture, is similar in frequency as the rest tremor but with lower amplitude, and is dopamine responsive. This appears to have a similar mechanism as the rest tremor. Pure postural tremor was present in 19% of the patients with postural tremor and was characterized by having a different frequency from the rest tremor, no pause on the assumption of posture, and not being dopamine responsive (Dirkx et al., 2018).

#### 3.3.6 Other specific tremor syndromes

Here we separately describe orthostatic tremor and functional (psychogenic) tremor because there are some differences in the methodology used for the tremor recording and analysis.

##### 3.3.6.1 Orthostatic tremor

Orthostatic tremor (OT) is a high frequency tremor (13–18 Hz) which manifests in the lower extremities (and sometimes upper extremities) when the patient stands up and attenuates when the patient walks (Hassan et al., 2016). In primary orthostatic tremor, the affected limbs oscillate at the exact same frequency suggesting only one central oscillator causes the tremor, which makes primary orthostatic tremor the only known non-functional tremor with left–right tremor coherence (Thompson et al., 1986, McAuley et al., 2000).

For the electrophysiological diagnosis, the number of EMG channels may be limited to two with surface electrodes placed on the bilateral tibialis anterior (TA) muscles and the EMG activity recorded with the patient sitting and standing. Typically, a 13–18 Hz frequency peak is observed on both TA muscles while standing only. Additional recordings of the surface EMG channels for the medial gastrocnemius should be considered if there are atypical features noted on the TA recordings. Atypical features may include a lower frequency of discharge and irregular bursting pattern which are characteristics of orthostatic myoclonus (OM). The frequency of OM is slower in the 3–7 Hz range with synchronous bursts commonly identified between homologous muscles, most commonly the TA. However, semi-rhythmic alternating bursts may be identified between the ipsilateral TA and medical gastrocnemius. These differences are distinguishable based only with the use of electrophysiology.

**Fig. 7:**
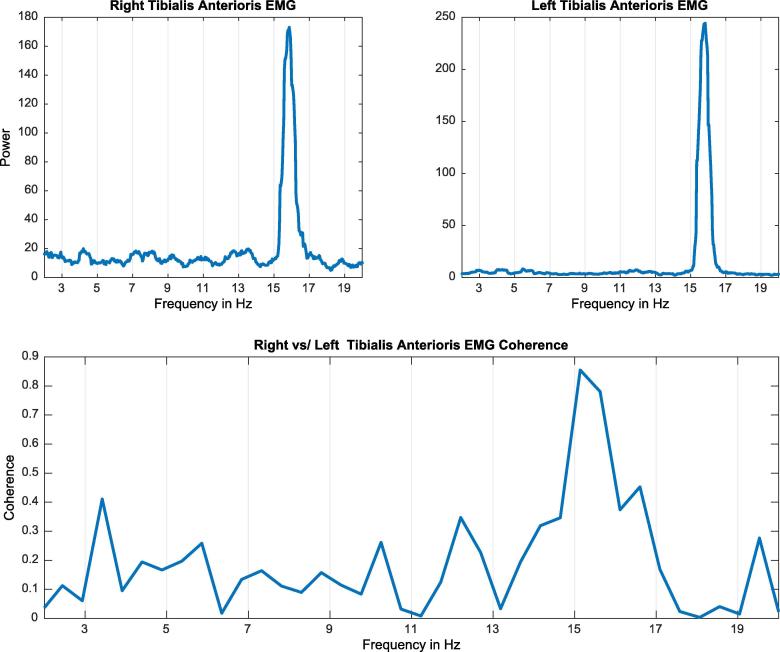
Example of orthostatic tremor. The plots on the top show an approximate 16 Hz peak on both tibialis anterior (TA) muscles when the patient is standing. The lower plot shows significant coherence between both tibialis anterior muscles at the frequency of the tremor.

The fact that both sides (left and right) are oscillating at the same frequency can be tested with coherence analysis between the signal of both extremities. In the case of OT patients, a very sharp peak at the same frequency of the tremor will be observed with coherence analysis (Fig. 7).

##### 3.3.6.2 Functional (psychogenic) tremor

Functional (psychogenic) tremor, similarly to most functional movement disorders, is characterized by irregularity and distractibility which are the two features to look for in a tremor study of these patients. The frequency usually ranges between 6 and 11 Hz for functional hand tremors (Brown and Thompson, 2001). In addition to recording the patient at rest, posture, posture plus loading and action, there are several other maneuvers that need to be done to help with the diagnosis.

Distractibility: The patient is asked to perform another task (can be a mental or a motor task) while the tremor is recorded. It is very important to record the spontaneous baseline tremor and then ask the patient to perform the task. After the patient finishes the task, it is recommended to continue the recording for a few more seconds in order to capture rebound of the tremor after completion of the task. Significant changes in the tremor pattern during the task, as compared to the pattern before and after the task, are suggestive of a functional origin of the tremor.

Entrainment: The patient taps with one hand at a frequency set by a metronome, while the tremor at the other hand is recorded. Entrainment occurs when the original tremor frequency in the affected limb shifts towards the tapping frequency. Asking patients to tap at 1.5, 2.0, and 2.5 Hz is typically sufficient to demonstrate entrainment in our experience. Functional tremor may entrain at the frequency at which the patient is tapping and there will be significant coherence between both EMG spectra at the tapping frequency (Schwingenschuh et al., 2016). It is important at the desired frequency to tap at a low amplitude to reduce the likelihood of mechanical transmission between the limbs which can erroneously be reported as coherence. For the same reason, the coherence is calculated between EMG channels and not between accelerometers. A failure of the patient to tap according to the instructions despite apparent ability to do so is also considered a sign of functionality. Thus, it is important to measure the patient’s tapping performance as well as the tremor. If the original frequency persists and there is a new peak at the frequency at which the patient is tapping, this finding can correspond to a mirror movement which is commonly observed in patients with dystonia and motor neuron disease (Merchant et al., 2018).

Ballistic movement: The patient is asked to perform a quick movement with one hand, for example, a fast wrist extension. During that hand’s movement, an interruption of the tremor in the contralateral hand (usually for more than 300 ms) is a sign compatible with functional tremor (Kumru et al., 2004).

Other signs that may be observed in functional tremor are irregularities in the tremor frequency and amplitude, an increase in the amplitude of the tremor when weights are added, or a short co-contraction of agonist-antagonist muscles when the tremor is starting (Deuschl et al., 1998).

Regarding the frequency domain analysis, in contrast to organic tremors (except for primary orthostatic tremor), functional tremor syndromes commonly demonstrate significant coherence between affected limbs (Raethjen et al., 2004). A functional tremor will never have a frequency as high as 13–18 Hz, thus there is no risk of confusing it with an orthostatic tremor. The coherence has to be calculated between EMG channels, as accelerometers are subject to co-oscillation due to pure mechanical transfer and are prone to over-estimation of coherence.

The different types of tremors and their electrophysiological characteristics are listed in Table 1.

**Table 1:** Types of tremors and their electrophysiological parameters.

	Frequency range	Components			EMG Left-Right coherence	Other
		Mechanical	Mechanical reflex	Central		
Physiological Tremor	6–12 Hz	Main component	No	May have a small 8–12 Hz	No	Usually asymptomatic
Enhanced Physiological Tremor	4–12 Hz	Main component	Main Component	May have a small 8–12 Hz	No	The main component may be mechanical or mechanical reflex
Essential tremor	4–12 Hz	Small component	No	Main Component	No	There is an inverse correlation between the frequency and age
Functional tremor	Variable, 6–11 Hz	Small component	No	Main Component	Sometimes	Variable and distractible
Orthostatic tremor	13–18 Hz	Small component	No	Main Component	Always	Only when standing
Parkinson tremor	4–6 Hz	Small component	No	Main Component	No	Two types of posture tremor, re-emergent (same frequency as rest) and no re-emergent.

## 4 Conclusions

The electrophysiological characterization of tremors can provide the clinician with very valuable information that is not possible to obtain from the physical examination and can facilitate the diagnostic and therapeutic approach to follow. Additionally, most of the equipment needed for the recordings are found in many clinical electrophysiology labs and at a relatively low cost, which can facilitate their use in the clinical setting.

Several clinical tremor syndromes overlap and therefore clinical correlation is always advisable. However, tremor analysis should not be considered just as an extension of a physical examination. Certain tremor syndromes such as OT can only be diagnosed based on electrophysiology. The clinical evaluation of functional tremors is often very complex, and electrophysiology is very useful in making the correct diagnosis. Tremor analysis can also serve as an objective test for diagnosing functional tremor which can facilitate patient counseling and acceptance of diagnosis by the patients which is a major hurdle in appropriate rehabilitation of these patients.

The diagnostic utility of electrophysiological tremor analysis in identifying organic tremors in patients diagnosed with psychiatric comorbidities and associated functional tremor is under recognized.

Identifying the presence of more than one etiology of the central component for tremor generation is another limitation of clinical assessment, but can be easily addressed by electrophysiological tremor analysis.

Although there are good arguments to implement this technique, the electrophysiological study of tremor is not widely available and in many cases is only done for research purposes. We believe that the clinical implementation of these techniques is critical to improve the diagnosis accuracy of tremors and should be a tool available to neurologists in the movement disorder field.
